# Dynamic Postural Control in Children: Do the Arms Lend the Legs a Helping Hand?

**DOI:** 10.3389/fphys.2018.01932

**Published:** 2019-01-17

**Authors:** Mathew W. Hill, Maximilian M. Wdowski, Adam Pennell, David F. Stodden, Michael J. Duncan

**Affiliations:** ^1^Centre for Sport, Exercise and Life Sciences, Coventry University, Coventry, United Kingdom; ^2^Human Performance and Development Laboratory, Department of Physical Education, University of South Carolina, Columbia, MO, United States

**Keywords:** balance regulation, upper extremities, arm movements, mobility, children

## Abstract

There is growing empirical evidence lending support to the existence of an “upper body strategy” to extend the ankle and hip strategies in maintaining upright postural stability among adults. Both postural stability and arm movement functions are still developing in children. Therefore, enquiry concerning arm contribution to postural stability among children is needed. This proof of concept study seeks to determine whether the arms play a functionally relevant role in dynamic postural control among children. Twenty-nine children (girls, *n* = 15; age, 10.6 ± 0.5 years; height, 1.48 ± 0.08 m; mass, 42.8 ± 11.4 kg; BMI, 19.2 ± 3.7 kg/m^2^) completed three dynamic balance tests; (1) Y Balance test^®^, (2) timed balance beam walking test, (3) transition from dynamic to static balance using the dynamic postural stability index (DPSI). Each test was performed with free and restricted arm movement. Restricting arm movements elicited a marked degradation in the Y Balance reach distance (all directions, *P* ≤ 0.001, *d* = -0.85 to -1.13) and timed balance beam walking test (*P* ≤ 0.001, *d* = 1.01), while the DPSI was the only metric that was not different between free and restricted arm movements (*P* = 0.335, *d* = -0.08). This study provides direct evidence that the arms play a functionally relevant role in dynamic balance performance among children. These findings may provide the impetus to develop training interventions to improve the use of the arms in activities of daily living.

## Introduction

The ability to maintain postural control plays an important role in child development, representing a fundamental pre-requisite to competently perform skilled movements and complex motor skills (Mickle et al., 2011; Verbecque et al., 2016). Although the etiology of falls is complex, maturational and/or experiential immaturities in static (i.e., increased postural sway) and dynamic (i.e., gait disorders, such as a loss of symmetry or slowing of gait speed) postural control have been identified as important intrinsic factors increasing the risk of falling and sustaining an injury in children (Khambalia et al., 2006; Granacher et al., 2011). Therefore, an improved understanding of postural control is important for the identification of children with an increased risk of falling and the development of fall-prevention interventions.

To maintain an upright position, the central nervous system (CNS) must continually integrate and (re)weigh information from visual, vestibular and proprioceptive systems to elicit coordinated muscular responses (Mickle et al., 2011). Nashner and McCollum (1985) proposed the existence of two distinct modes of operation to maintain upright posture, referred to as the ankle and hip strategies. Mechanically, the ankle strategy is predominantly used during slow and small amplitude perturbations (i.e., quiet bipedal standing) by moving the whole body as a single segment inverted pendulum controlled by ankle joint torque (Runge et al., 1999). In contrast, the hip strategy, which moves the body as a double-segment inverted pendulum with counterphase motion at the ankle and hip, is expected to be employed for fast or large amplitude perturbations, or when the support surface is narrow so that only little ankle torque can be applied can be applied (Horak and Nashner, 1986). However, it has been suggested that postural control is multivariate (as opposed to bivariate) in nature (Hsu et al., 2007; Kilby et al., 2015). Although an upright posture can usually be maintained by the ankle and hip during most scenarios, movements of the upper body are not taken into account by these two control strategies. Indeed, most clinical balance and mobility tests do not evaluate arm movements. Although many studies acknowledge that the arms play a major role in maintaining dynamic postural control (Bruijn et al., 2010; Sawers and Ting, 2015), some studies allow free arm movements (Faigenbaum et al., 2014), others restrict arm movement (Muehlbauer et al., 2013), while others do not provide specific details of arm position (Geldhof et al., 2006; Humphriss et al., 2011). Given these observations, it is important to investigate the potentially important role of arm movements on postural stability, which will be valuable in elucidating some of the fundamental aspects of postural control development in children.

There is growing empirical evidence lending support to the existence of an “upper body strategy” to enhance the body of work that includes ankle and hip postural stability strategies (Hsu et al., 2007), particularly during challenging dynamic tasks (e.g., walking across a narrow beam) (Boström et al., 2018). Accordingly, the influence of arm movements on postural control only appears to be evident underchallenging constraints or task demands. For example, restricting arm movements impairs performance in functional mobility tests (i.e., timed-up and-go) (Milosevic et al., 2011), reduces dynamic postural control (i.e., Y Balance test) (Hébert-Losier, 2017) and impairs mechanisms to minimize postural sway during quiet tandem standing (Patel et al., 2014). In addition, arm movements play a functional role in postural recovery during standing (Allum et al., 2002; Maki and McIlroy, 2006) and walking (Marigold et al., 2003; Roos et al., 2008; Pijnappels et al., 2010), further strengthening the hypothesis that the upper extremities play an important role during challenging postural tasks. Therefore, it is reasonable to suggest that the contribution of the arms becomes important after a certain “threshold” of postural stress has been met.

The demands placed on the postural control system during dynamic postural tasks (i.e., walking across a narrow beam or standing on a single limb and reaching with the contralateral limb), are considerably greater than standing or walking (Boström et al., 2018). Although evidence has demonstrated a degradation in postural control with restricted arm movements in young (Patel et al., 2014; Hébert-Losier, 2017; Boström et al., 2018) and intermediate aged (Milosevic et al., 2011) adults, little is known about the role of arm movements on dynamic postural control in children. Indeed, there is a reasonable theoretical basis for expectation that arm movements will make a substantial and functionally relevant contribution to dynamic postural tasks in children, because their neuromuscular system is not yet fully matured and fundamental motor skills are still emerging (Granacher and Gollhofer, 2012). Determining whether arm movements play a role in performance of dynamic postural control tasks will be influential in guiding future efforts to incorporate/exclude upper body movements into rehabilitation, training and assessment protocols designed to test and improve postural control in children. These findings will also be influential in providing the impetus for future research to clearly define and describe arm placement and movement to avoid misinterpretation of dynamic postural tasks and for replication purposes.

Therefore, the aim of this study was to investigate the effects of arm movements on the performance of dynamic postural tasks in children. Considering the important contribution of arm movements to dynamic postural control in adults (Milosevic et al., 2011; Patel et al., 2014; Hébert-Losier, 2017) we hypothesize that free arm movements lead to better postural performance among children than restricted arm movements. The difference in performance between restricted and non-restricted conditions will provide a specific quantitative assessment of individuals reliance on lower body postural control mechanisms.

## Materials and Methods

### Participants

An *a priori* power analysis (statistical power = 0.80, alpha = 0.05, effect size = 0.48) was conducted for composite Y Balance score (Hébert-Losier, 2017) and revealed that 29 participants would be sufficient for finding statistically significant effects of arm restriction on dynamic balance performance. Thus, the present study consisted of a group of twenty-nine (girls, *n* = 15; age, 10.6 ± 0.5 years [range, 10.1–11.2 years]; height, 1.48 ± 0.08 m; mass, 42.8 ± 11.4 kg; BMI, 19.2 ± 3.7 kg/m^2^, waist circumference, 68.6 ± 9.1 cm; right foot dominant, *n* = 27; dominant leg length, 79.0 ± 5.3 cm) children, recruited from their primary schools in the city of Coventry, United Kingdom. Foot dominance was defined as the foot used to kick a ball. Physical Maturity was assessed by predicting the age at peak height velocity (APHV) using the equation (Mirwald et al., 2002), which is a method based on the growth patterns of the upper body and legs of every individual and is compared to the average population with the aim to classify children between early, average and late maturers. This technique of measuring APHV was chosen because it had the advantages of being non-invasive and more economical in relation to labor and monetary cost (Mirwald et al., 2002). All parents completed a health screen questionnaire prior to participation. This requested information relating to any physical, cognitive or other issues that prevented participation in physical activity. This includes details in relation to chronic disease (e.g., diabetes), special educational needs (e.g., ADHD), injuries, muscular deficits or cardiovascular impairments as well as confirming that children had normal vision and no auditory impairments. Following institutional ethics approval and prior to conducting the experiment, all participants as well as the children’s parents gave their written informed consent. The study was carried out in accordance with the guidelines outlined in the Declaration of Helsinki (1964).

### Experimental Procedure

Participants completed dynamic postural tasks of varying difficulty under two different verbally conveyed instructions of arm position; (1) arms placed flat across the chest touching the contralateral shoulder (i.e., restricted arm movement) and (2) arm movement without restriction (i.e., free arm movement). To ensure familiarization and to remove potential learning effects, each participant completed three practice trials and three recorded trials for each test condition (i.e., arms vs. no-arms) The order of balance tasks was randomized, as were the arm position instructions. For the free arm movement, participants were instructed to be able to move their arms freely during the tasks. For the restricted arm position, compliance to the instructions was monitored visually by the investigators. If the arms moved away from the chest the trial was discarded and repeated. Given the age of the participants, minor arm adjustments were permitted. The investigators were always available to assist the participants to complete the tests safely.

### Y Balance Test

The Y Balance Test Kit^TM^ was used to determine dynamic postural control. As described by Plisky et al. (2006), the Y Balance Test Kit^TM^ consists of a stance platform to which three pieces of plastic pipe are attached in the anterior, posteromedial, and posterolateral reach directions. The posteromedial and posterolateral pipes are positioned 135° from the anterior pipe with 45° between the posterior pipes. Participants stood on the center of a foot plate with the most distal point of the great toe at the starting line. While maintaining a single-leg stance with the dominant limb, participants were asked to push a target (reach indicator) along the pipe with the contralateral limb (i.e., non-dominant limb) in the anterior, posteromedial and posterolateral directions. Maximal reach distance was measured by reading the tape measure at the edge of the reach indicator, reflecting the point where the most distal part of the foot reached. The trial was discarded and repeated if the participant (1) failed to maintain single limb stance (i.e., touch the floor with the reach limb), (2) failed to remain in contact with the reach indicator at the most distal point (i.e., kicked the reach indicator to achieve greater distance), (3) used the reach indicator to support weight (i.e., mechanical support) or (4) failed to return to the reach foot at the center of the foot plate. Although the reach direction was randomized, to improve reproducibility of the testing protocol, participants performed three consecutive reach attempts for each direction. The greatest reach distance for each direction was used for subsequent analysis. Reach distance was normalized to limb length (reach distance/limb length ^∗^ 100) (Plisky et al., 2006). Each participant’s dominant limb length was measured in centimeters from the anterior superior iliac spine to the most distal portion of the medial malleolus using an anthropometric measuring tape (Gribble and Hertel, 2003). Additionally, the composite reach score was also calculated as the sum of the three reach distances divided by three times the limb length and multiplied by 100. A composite reach score was calculated as the sum of the three reach directions divided by three times limb length, and then multiplied by 100 (Plisky et al., 2006). A composition score below 94% is related to neuromotor deficit and a greater probability of injuries (Plisky et al., 2006). Therefore, we used this criterion to determine the clinical relevance of changes in Y Balance performance with restricted arm movements.”

### Dynamic Postural Stability Index

Dynamic postural stability index (DPSI) was assessed using an anterior jump-landing task on the dominant limb (Sell, 2012). DPSI is a unitless composite score of anteroposterior (*y*), mediolateral (*x*), and vertical (*z*) ground reaction forces (GRF) and is similar to the static postural stability task, in that a higher DPSI indicates worse postural control (Sell, 2012). Participants were instructed to stand on two legs at distance of 40% of their body height from the center of the force platform (AMTI, AccuGait, Watertown, MA, United States). Each participant was instructed to jump forward over a 6-inch hurdle on to the force platform and land on their dominant limb, stabilize as quickly as possible and, balance for 10 s. The hurdle was positioned at a distance of 20% of their body height (i.e., half way between the starting point and the center of the force platform). Each participant completed a minimum of three practice attempts. Trials were discarded and repeated if the participants contralateral limb touched the floor. Data were sampled at 200 Hz (AMTI, Netforce, Watertown, MA, United States) and data were passed through a fourth order low pass Butterworth filter with a 20 Hz cut-off frequency. DPSI was calculated using the first 3 s of the ground reaction forces following initial contact, defined as the instant the vertical ground reaction force exceeded 15 N (equation below). An average DPSI from the three trials in each condition was used for further analysis.

$$DPSI=\left(\sqrt{\frac{\sum {\left(GRFx\right)}^{2}+\sum {\left(GRFy\right)}^{2}+\sum {\left(body\;weight-GRFz\right)}^{2}}{number\;of\;data\;points}}\right)$$

### Tandem Walk

For the tandem walk test, participants were asked to walk along a 2-m length balance beam (8 cm width), starting with the dominant foot, and complete three measurements for each arm position. The width of the beam was chosen based on previous research (Sawers and Ting, 2015) and prior feasibility testing. All participants wore comfortable shoes. Walking speed was self-selected, but participants were aware they were being timed. The time taken to step on to the balance beam, walk along the balance beam, step off, turn around, step back onto the balance beam and return back to the original position was recorded in seconds by two raters using a stopwatch. Task failure was defined as stepping off the beam during the trial. For safety, two members of staff walked either side of the participant to prevent falling, but without interfering with the test. The fastest times for free and restricted arm movements were used in subsequent analysis.

### Data Analysis

Data were analyzed using SPSS version 24.0 (IBM Inc., Chicago, IL, United States). Paired *t*-tests were carried out to determine differences in dynamic balance and mobility performance between free arm and restricted arm movements. For all analyses, normality (Shapiro–Wilk test) and homogeneity of variance/sphericity (Levene’s test) were performed and confirmed prior to parametric analyses. Data were also analyzed for practical meaningfulness using magnitude-based inferences. Magnitude of effect size (Cohen’s *d*) was calculated for all metrics and were interpreted using thresholds of ≤0.2 (trivial), 0.2 (small), 0.6 (moderate), 1.2 (large), and 2.0 (very large) (Hopkins et al., 2009). Statistical significance was accepted at *P* ≤ 0.05.

## Results

**FIGURE 1:**
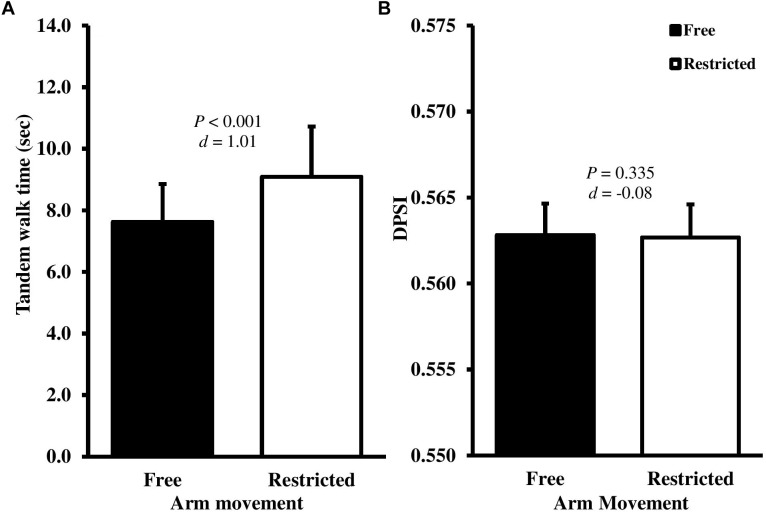
Mean ± SD tandem walk time **(A)** and dynamic postural stability index (DPSI) **(B)** with free (black bars) and restricted (white bars) arm movements. *d*-Value represents Cohen’s *d* effect size.

**FIGURE 2:**
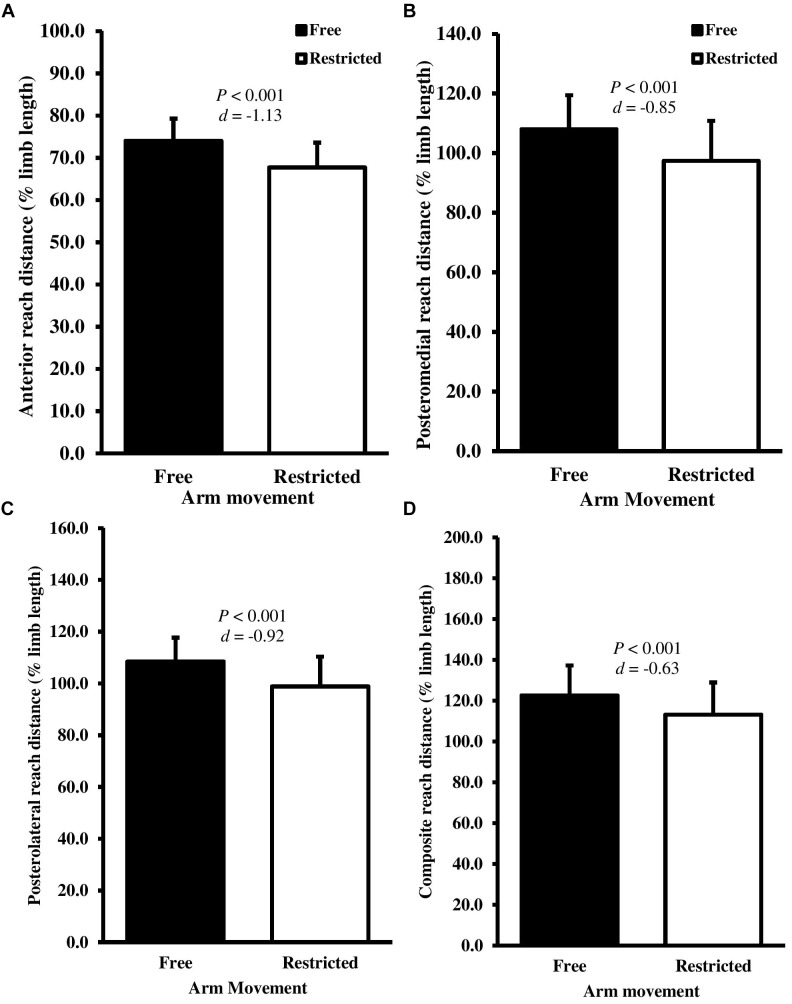
Mean ± SD normalized **(A)** anterior, **(B)** posteromedial, **(C)** posterolateral, and **(D)** composite reach distance for the Y Balance test with free (black bars) and restricted (white bars) arm movements. *d*-Value represents Cohen’s *d* effect size.

Recognizing that gender and maturation status may influence the performance of balance assessments, as part of our initial exploratory analyses we conducted a 2 (*gender*; male and female) × 2 (*arm contribution*; free and restricted) way analysis of co-variance (ANCOVA), controlling for APHV to determine the effects of gender as a between-subject factor. There were no significant interactive or main effects of gender for any of the outcome measures. Arm movement had the greatest effect in the balance beam test (Table 1). Mean balance beam walking time increased by 1.5 s (19.2%) when participants arm movements were restricted (*t*_(28)_ = -10.889, *P* < 0.001, *d* = 1.01) (Figure 1A). In contrast, the DPSI did not show statistically significant changes with restricted arm movement (*t*_(28)_ = 0.940, *P* = 0.335, *d* = -0.08) (Figure 1B). The Y Balance reach distance decreased in the anterior (mean diff; 6.3 cm, *t*_(28)_ = 11.563, *P* < 0.001, *d* = -1.13), posteromedial (mean diff; 9.6 cm, *t*_(28)_ = 6.627, *P* < 0.001, *d* = -0.85) and posterolateral (mean diff; 10.6 cm *t*_(28)_ = 8.653, *P* < 0.001, *d* = -0.92) directions when arm movements were restricted. Accordingly, composite Y Balance score also decreased by 9.6% (*t*_(28)_ = 7.638, *P* < 0.001, *d* = -0.63) (Figures 2A–C). Although none of the participants were classified as at-risk with free arm movements, four participants were identified as at-risk when the arms were restricted (Figure 2D).

**Table 1:** Mean ± SD and Cohen’s *d* effects size for the difference in dynamic balance performance between free and restricted arm movement conditions.

Variable	Free-arm movement	Restricted arm-movement	Cohen’s *d*
Tandem walk (sec)	7.6 ± 1.2	9.1 ± 1.6^*^	1.01
Dynamic postural stability index	0.563 ± 0.002	0.563 ± 0.002	-0.08
Y Balance test anterior direction (% leg length)	74.0 ± 5.3	67.7 ± 5.9^*^	-1.13
Y Balance test posteromedial (% leg length)	108.4 ± 9.3	98.8 ± 11.6^*^	-0.92
Y Balance test posterolateral (% leg length)	107.9 ± 11.3	97.7 ± 13.3^*^	-0.83
Composite score (% leg length)	122.5 ± 14.7	113.1 ± 15.8^*^	-0.62


*^∗^Indicates significant difference between free and restricted arm conditions (*P* < 0.001).*

## Discussion

This is the first study to examine the effect of the use of the arms on multiple measures of dynamic postural control in a pediatric population. The results are therefore novel and have practical application for physical therapists, sport and exercise scientists, physical educators and strength and conditioning coaches who work with children to enhance movement performance. We found moderate to large magnitude reductions in the performance of two out of three dynamic postural control tests when arm movements were restricted, partially supporting our hypothesis. The results of the present study suggest that arm movements significantly influence performance in dynamic postural situations in children. Such findings align with prior work conducted in adults which suggests the existence of an “upper body strategy” (Hsu et al., 2007; Boström et al., 2018). These important findings also indicate that upper body movements should be incorporated into assessment protocols designed to test and improve postural control in children. More specifically, arm movements could be standardized (i.e., arms stretched out at an angle of 90° shoulder abduction in the frontal plane) or completely restricted (i.e., hands across chest).

Previous research has indicated that a composite Y Balance reach score of less than or equal to 94% was significantly associated with lower extremity injury (Plisky et al., 2006). As expected, a significant degradation in reach distance was observed when arm movements were restricted. Although only four participants fell below the 94% criterion line with restricted arm movements, all participants demonstrated a reduction in reach distance (Δ-2 to -22%), indicating a general decline in dynamic postural control. Although the result of the Y Balance test are not intended to infer an increased risk of injury, this study seeks to evaluate the extent to which the arms contributed (or not) to performance in dynamic tasks in children, as no study to date had examined this in children. This is an important first step before additional exploration could be undertaken in relation to injury risks. Importantly, we found that anterior reach distance was the most affected direction with restricted arm movements. These findings are in direct contrast to previous reports in young adults, where anterior reach distance did not differ between free and restricted arm movements (Hébert-Losier, 2017). Anterior reach with the lower limb involves posterior displacement of body mass away from the base of support, which is an uncommon task (i.e., upper body leaning backward). In contrast, posterior reach directions require the body mass to be displaced anteriorly (i.e., upper body reaching forward), which is more functionally relevant and familiar to children. Therefore, the posteromedial and posterolateral reach directions may utilize more practiced motor patterns and are thus less susceptible to change than anterior reach seen here. The most likely explanation for the better reach performance in the free arm movement condition relates to the mechanical effects of outstretching the arms. Specifically, greater dispersion of body mass in the sagittal plane from a vertical line perpendicular from the base of support increases the moment of inertia, which should theoretically increase stability of the postural control system. Additionally, free arm movements may generate restoring torque to aid dynamic postural control (Patel et al., 2014).

Data indicated the timed beam walk was suitable as a quantitative assessment of dynamic stability based on its sensitivity and discriminatory capability in this sample. The timed tandem beam walk test lacks predictive validity/diagnostic cut-off values, therefore, the 1.5 s mean difference in performance is harder to practically interpret. However, this finding suggests that the arms beneficially contribute to one or more of the tasks of stepping up, stepping down, turning or walking across a narrow beam. With respect to the later, Boström et al. (2018) recently examined movements of the upper and lower body during tandem beam walking. They reported that when the task became more difficult (i.e., for narrower beam width), the contribution of upper body movements to balance maintenance increased, while the lower body contribution remained the same. Taken together, these findings suggest that the arms hierarchically compliment the lower body during dynamic balance scenarios.

In the present study, the DPSI was the only postural control metric which did not show significant changes with restricted arm movement. This finding was not expected. Outstretching the arms has been shown to reduce postural sway during quiet tandem standing (Patel et al., 2014). It is possible that ankle, knee and hip neuromuscular response strategies would effectively respond to the directionality of this task (i.e., anterior jump). Thus, improvements in performance with the arms stretched out to the side may only be evident under task constraints which challenge postural control in the frontal plane (i.e., tandem walk, or lateral jumps) (Milosevic et al., 2011; Patel et al., 2014; Hébert-Losier, 2017). A further possibility for the non-significant improvement in DPSI seen here reflects a “ceiling effect” in the restricted arm movement condition. Specifically, the task of jumping forward and landing on a single limb may have already been close to optimal, and therefore using the arms freely does now allow for any noticeable improvement in balance. Further, much like the Y Balance test and the tandem walk, the DPSI is quantitative in nature. Therefore, it is possible that postural control strategies helped absorb the vertical ground reaction force from contact to stabilization of the vertical displacement of the COM during landing.

### Implications

There are several important implications to be garnered from the present study. Firstly, this study provides the first direct evidence that the arms play a functionally relevant role in certain dynamic postural tasks among children. In agreement with previous research (Milosevic et al., 2011; Hébert-Losier, 2017) we support the recommendations that future research should clearly define and describe arm placement and movement to avoid misinterpretation of dynamic balance tests and to facilitate experimental replication. Similarly, studies that adopt either a restricted or free arm movement should not be used interchangeably. The intent of this study was not to simply provide recommendations for arm placement, as this will depend upon the aims of the clinician/therapist. Instead, this proof of concept study intended to elucidate the importance of the use of the arms as a critical factor in postural stability strategies to better inform clinicians, physical therapists, researchers and practitioners for the purposes of identifying impairments, planning individualized interventions and evaluating change over time. We suggest that permitting arm movements is more functionally relevant to typical activities of daily living, but it is difficult to control the variability and dynamic nature of how individuals use the arms. In contrast, restricting arm movements is likely to provide a more definite and standardized assessment of lower limb function. More specifically, this task controls for the differential use of the arms to overcome a lack of postural control demonstrated by the ankle, knee, and hip postural control mechanisms. Thus, assessing the difference in performances scores during restricted and non-restricted protocols may provide a better understanding of the extent to which people use their arms to further improve balance even when their lower body postural control is well-developed. These findings might also provide the impetus to develop training interventions to enhance postural control by employing constraint-based strategies with the arms in activities of daily living. Specifically, the observed improvement in performance with free arm movement suggests that initially allowing arm movements may be valuable in acting as a starting point as part of a continuum of balance training to progress to more challenging programs (i.e., restricted arm movements). In contrast, it may also be appropriate to restrict arm movements to decrease the moment of inertia to promote more effective control of the COM by focusing on ankle, knee and hip coordinative development strategies. Such proximal-distal strategies may promote a more sensitive anticipatory and/or recovery postural response mechanism.

### Limitations

A few limitations in this work should be acknowledged. Firstly, we were unable to measure or control arm movements (i.e., kinematics) during either of the free or restricted conditions. As no quantitative movement analysis was undertaken we are aware that this study cannot comprehensively contribute to understanding upper body strategies used for movement. We are conscious that, due to the demands placed on participants and their age, we were unable to also measure ankle, knee, and hip postural control mechanisms. Future research would, however, be welcome which addresses this issue. Secondly, we calculated the DPSI from a forward jump. Given the important contribution of arm movements to lateral postural control, future studies should examine the effects of arm movement on a lateral jump (Wikstrom et al., 2005; Sell, 2012). A more detailed analysis of dynamic stability should also include the anteroposterior and mediolateral stability index (Wikstrom et al., 2006). We did not calculate the mediolateral stability index because it has previously been shown to have poor test-retest reliability (*r* = 0.38) and a high standard error of measurement as a percentage of the mean score (26.1%) compared to the DPSI (3.7%) (Wikstrom et al., 2005). Another limitation was that we only examined Y Balance test performance on the dominant limb (stance limb). Therefore, we are precluded from calculating asymmetry between the dominant and non-dominant leg. It is likely that the detrimental effects of arm restriction on Y Balance test performance would be more pronounced on the non-dominant leg. Finally, we did not measure reactive balance. Several studies have reported that arm movements play a functional role in trip recovery in response to perturbations during standing (Allum et al., 2002; Maki and McIlroy, 2006) and walking (Marigold et al., 2003; Roos et al., 2008; Pijnappels et al., 2010) scenarios. It is likely that the arms serve as a counterweight to shift the body COM away from the direction of the fall (Marigold et al., 2003) or generate restoring torque to reduce angular momentum of the body (Roos et al., 2008). Therefore, it is likely that the contribution of arm movements in children may be even greater during reactive balance tasks. Future research should examine upper limb dynamics in the recovery phase of a perturbation during standing and locomotion.

## Conclusion

In summary, this is the first study to demonstrate evidence of a marked degradation in the performance of the balance beam walking and Y Balance tests when arm movements are restricted among children. Overall, it appears feasible for clinicians and practitioners to test the effectiveness of arm usage in balance and mobility tasks by calculating difference scores between restricted and non-restricted arm conditions. The results highlight that the difference score between the restricted and non-restricted conditions provide a robust quantitative assessment that may identify individuals that rely more heavily on lower limb postural stability mechanisms. Future research should expand on these findings by exploring which individuals rely most on arm movements for postural control (i.e., potential moderator variables).

## Author Contributions

MH, MW, AP, and MD conceived and designed the research. MH, AP, and MW conducted the experiments. MH performed the analyses and wrote the manuscript. AP, MD, MW, and DS revised the manuscript. All authors read and approved the final manuscript.

## Conflict of Interest Statement

The authors declare that the research was conducted in the absence of any commercial or financial relationships that could be construed as a potential conflict of interest.

## References

[B1] Allum J. H. J. Carpenter M. G. Honegger F. Adkin A. L. Bloem B. R. (2002). Age-dependent variations in the directional sensitivity of balance corrections and compensatory arm movements in man. *J. Physiol.* 542 643–663. 10.1113/jphysiol.2001.015644 12122159 PMC2290411

[B2] Boström K. J. Dirksen T. Zentgraf K. Wagner H. (2018). The contribution of upper body movements to dynamic balance regulation during challenged locomotion. *Front. Hum. Neurosci.* 12:8. 10.3389/fnhum.2018.00008 29434544 PMC5790866

[B3] Bruijn S. M. Meijer O. G. Beek P. J. van Dieën J. H. (2010). The effects of arm swing on human gait stability. *J. Exp. Biol.* 213 3945–3952. 10.1242/jeb.045112 21075935

[B4] Faigenbaum A. D. Myer G. D. Fernandez I. P. Carrasco E. G. Bates N. Farrell A. (2014). Feasibility and reliability of dynamic postural control measures in children in first through fifth grades. *Int. J. Sports Phys. Ther.* 9 140–148. 24790775 PMC4004119

[B5] Geldhof E. Cardon G. De Bourdeaudhuij I. Danneels L. Coorevits P. Vanderstraeten G. (2006). Static and dynamic standing balance: test-retest reliability and reference values in 9 to 10 year old children. *Eur. J. Pediatr.* 165 779–786. 10.1007/s00431-006-0173-5 16738867

[B6] Granacher U. Gollhofer A. (2012). Is there an association between variables of postural control and strength in prepubertal children? *J. Strength Cond. Res.* 26 210–216. 10.1519/JSC.0b013e31821b7c30 22201695

[B7] Granacher U. Muehlbauer T. Gollhofer A. Kressig R. W. Zahner L. (2011). An intergenerational approach in the promotion of balance and strength for fall prevention–a mini-review. *Gerontology* 57 304–315. 10.1159/000320250 20720401

[B8] Gribble P. A. Hertel J. (2003). Considerations for normalizing measures of the star excursion balance test. *Meas. Phys. Educ. Exerc. Sci.* 7 89–100. 10.1207/S15327841MPEE0702_3

[B9] Hébert-Losier K. (2017). Clinical implications of hand position and lower limb length measurement method on Y-balance test scores and interpretations. *J. Athl. Train.* 52 910–917. 10.4085/1062-6050-52.8.02 28937801 PMC5687235

[B10] Hopkins W. G. Marshall S. W. Batterham A. M. Hanin J. (2009). Progressive statistics for studies in sports medicine and exercise science. *Med. Sci. Sports Exerc.* 41 3–13. 10.1249/MSS.0b013e31818cb278 19092709

[B11] Horak F. B. Nashner L. M. (1986). Central programming of postural movements: adaptation to altered support-surface configurations. *J. Neurophysiol.* 6 1369–1381. 10.1152/jn.1986.55.6.1369 3734861

[B12] Hsu W. L. Scholz J. P. Schoner G. Jeka J. J. Kiemel T. (2007). Control and estimation of posture during quiet stance depends on multijoint coordination. *J. Neurophysiol.* 97 3024–3035. 10.1152/jn.01142.2006 17314243

[B13] Humphriss R. Hall A. May M. Macleod J. (2011). Balance ability of 7 and 10 year old children in the population: results from a large UK birth cohort study. *Int. J. Pediatr. Otorhinolaryngol.* 75 106–113. 10.1016/j.ijporl.2010.10.019 21074865

[B14] Khambalia A. Joshi P. Brussoni M. Raina P. Morrongiello B. Macarthur C. (2006). Risk factors for unintentional injuries due to falls in children aged 0–6 years: a systematic review. *Inj. Prev.* 12 378–381. 10.1136/ip.2006.012161 17170185 PMC2564414

[B15] Kilby M. C. Molenaar P. C. Newell K. M. (2015). Models of postural control: shared variance in joint and COM motions. *PLoS One* 10:e0126379. 10.1371/journal.pone.0126379 25973896 PMC4431684

[B16] Maki B. E. McIlroy W. E. (2006). Control of rapid limb movements for balance recovery: age-related changes and implications for fall prevention. *Age Ageing* 35 ii12–ii18. 10.1093/ageing/afl078 16926197

[B17] Marigold D. S. Bethune A. J. Patla A. E. (2003). Role of the unperturbed limb and arms in the reactive recovery response to an unexpected slip during locomotion. *J. Neurophysiol.* 89 1727–1737. 10.1152/jn.00683.2002 12611998

[B18] Mickle K. J. Munro B. J. Steele J. R. (2011). Gender and age affect balance performance in primary school-aged children. *J. Sci. Med. Sport* 14 243–248. 10.1016/j.jsams.2010.11.002 21276751

[B19] Milosevic M. McConville K. M. V. Masani K. (2011). Arm movement improves performance in clinical balance and mobility tests. *Gait Posture* 33 507–509. 10.1016/j.gaitpost.2010.12.005 21227695

[B20] Mirwald R. L. Baxter-Jones A. Bailey D. A. Beunen G. P. (2002). An assessment of maturity from anthropometric measurements. *Med. Sci. Sports Exer.* 34 689–694. 10.1097/00005768-200204000-00020 11932580

[B21] Muehlbauer T. Besemer C. Wehrle A. Gollhofer A. Granacher U. (2013). Relationship between strength, balance and mobility in children aged 7–10 years. *Gait Posture* 37 108–112. 10.1016/j.gaitpost.2012.06.022 22832473

[B22] Nashner L. M. McCollum G. (1985). The organization of human postural movements: a formal basis and experimental synthesis. *Behav. Brain Sci.* 8 135–150. 10.1017/S0140525X00020008

[B23] Patel M. Buckwell D. Hawken M. Bronstein A. M. (2014). Does outstretching the arms improve postural stability? *Neurosci. Lett.* 579 97–100. 10.1016/j.neulet.2014.07.010 25038417

[B24] Pijnappels M. Kingma I. Wezenberg D. Reurink G. van Dieën J. H. (2010). Armed against falls: the contribution of arm movements to balance recovery after tripping. *Exp. Brain Res.* 201 689–699. 10.1007/s00221-009-2088-7 19949781

[B25] Plisky P. J. Rauh M. J. Kaminski T. W. Underwood F. B. (2006). Star excursion balance test as a predictor of lower extremity injury in high school basketball players. *J. Orthop. Sports Phys. Ther.* 36 911–919. 10.2519/jospt.2006.2244 17193868

[B26] Roos P. E. McGuigan M. P. Kerwin D. G. Trewartha G. (2008). The role of arm movement in early trip recovery in younger and older adults. *Gait Posture* 27 352–356. 10.1016/j.gaitpost.2007.05.001 17561398

[B27] Runge C. F. Shupert C. L. Horak F. B. Zajac F. E. (1999). Ankle and hip postural strategies defined by joint torques. *Gait Posture* 10 161–170. 10.1016/S0966-6362(99)00032-6 10502650

[B28] Sawers A. Ting L. H. (2015). Beam walking can detect differences in walking balance proficiency across a range of sensorimotor abilities. *Gait Posture* 41 619–623. 10.1016/j.gaitpost.2015.01.007 25648493

[B29] Sell T. C. (2012). An examination, correlation, and comparison of static and dynamic measures of postural stability in healthy, physically active adults. *Phys. Ther. Sport* 13 80–86. 10.1016/j.ptsp.2011.06.006 22498148

[B30] Verbecque E. Vereeck L. Hallemans A. (2016). Postural sway in children: a literature review. *Gait Posture* 49 402–410. 10.1016/j.gaitpost.2016.08.003 27505144

[B31] Wikstrom E. A. Arrigenna M. A. Tillman M. D. Borsa P. A. (2006). Dynamic postural stability in subjects with braced, functionally unstable ankles. *J. Athl. Train.* 41 245–250. 17043691 PMC1569562

[B32] Wikstrom E. A. Tillman M. D. Smith A. N. Borsa P. A. (2005). A new force-plate technology measure of dynamic postural stability: the dynamic postural stability index. *J. Athl. Train.* 40 305–309. 16404452 PMC1323292

